# Physicians’ leadership styles in rural primary medical care: How are they perceived by staff?

**DOI:** 10.3109/02813432.2013.874083

**Published:** 2014-03

**Authors:** Jan Hana, Rudi Kirkhaug

**Affiliations:** ^1^Faculty of Health Sciences, National Centre for Rural Medicine, University of Tromsoe, Norway; ^2^Faculty of Humanities, Social Sciences and Education, University of Tromsoe, Norway

**Keywords:** Context, general practice, lead physicians, leadership style, Norway, rural medicine, staff gender, staff maturity, staff profession

## Abstract

*Aim.* This study investigates which leadership styles can be identified among general practice lead physicians and how they are associated with and predicted by staff and context characteristics like profession, gender, age, work experience, and team size. *Method/material.* In a cross-sectional study self-administered questionnaires were distributed to staff physicians (42% females) and support staff (98% females) at 101 primary health care centres in North Norway. A total of 127 and 222, respectively, responded (response rate 59%). Items were ranked on Likert scales (range 1–5). *Results*. Analysis revealed three significantly different styles (mean scores/Cronbach's alpha): change style (3.36/0.898), task style (3.17/0.885), and relation style (2.88/0.900). The lead physicians were perceived as practising change style the most and relation style the least. Males experienced significantly more of all three styles. Support staff scored lowest for all styles. Age was negatively correlated with relation style and change style, while work experience was negatively correlated with change style. No significant association was found between styles and team size. *Conclusion*. Leadership in rural general practice can be identified in terms of task, relation, and change styles. Change style is the most perceived style. Males seem to be most attentive to leadership styles. However, within the staff physician group, there is less difference between genders. Support staff scores lowest for all styles; this might indicate either less need for leadership or dissatisfaction with leadership. Age and work experience seem to reduce employees’ attention to relation and change styles, indicating that maturity reduces needs for these leadership styles. Due to growing demands for leaders to take care of efficiency and change in general practice, more young female physicians, and more diverse staff groups, these findings may be useful to understand leadership and leadership training for general practice.

Leadership styles practised by lead physicians in rural general practice are poorly investigated. This study has revealed that:

- Styles reflect general theory but are modified in PHC organizations.
- The leadership style “Change style” is most practised.
- Male staff are most attentive to leadership.
- Support staff experience least of all styles.

Staff maturity, such as age and experience, reduces staff's need for leadership.

## Introduction

It is generally accepted that leadership is a critical factor in organizations as it may affect goals, visions, strategy, social environment, and work motivation among employees [1]. This understanding also seems to be gaining acceptance in primary health care, as national and international health policy documents underline the importance of leadership for development of care [2–4]. In Norway both strategic and frontline leadership have been emphasized, but leadership training for medical undergraduates has been limited. The specialities in general practice and community medicine require two and six days of leadership training, respectively [5]. However, rural lead physicians seem to regard this training as inadequate, and instead rely on their clinical training as to relational and problem-solving competence [6]. However, data informing which leadership styles should be developed are still missing.

Primary medical care in Norway is organized by teams of physicians and support staff in health centres/doctors’ stations. Generally, leadership is the responsibility of a physician who has a merged clinical and public health position (mixed lead) [7]. At the time of this study, the mixed lead positions were dominated by males (78%) [8].

The fourfold goal of this study is to reveal which leadership styles exist among lead physicians, how dominant they are, how these styles are distributed among professions and genders, and how they are associated with and predicted by staff and context characteristics. Revealing data on these issues may help primary medical care to develop adequate leadership training.

In order to meet this goal we sought theoretical support in classical leadership theory defining leadership as an attributed phenomenon involving intended influence over other people to guide, structure, and facilitate activities and relationships in a group or organization [1,18]. Leadership is thus not only characteristics objectively held by the leader, but also conceptions and expectations in the heads of the followers. In accordance with such a definition, we rely on a contingency approach in order to identify which style is dominant and in demand. Classical leadership theory presents three major leadership styles [1,9,10]. First, the *task style* focuses on goal setting, distribution of tasks, coordination, monitoring, and rewarding. Second, the *relation style* is occupied with motivation, defining visions, coaching, support of individual development, and social maintenance. Finally, the *change style* focuses on managing organizational change processes through seeking different perspectives and encouraging new ways of improving services.

The contingency approach advocates that leadership style may be due not only to the leader's personal and professional qualifications, but as much to the conditions the leader faces in practising leadership [1,11,12]. Therefore, we expected that staff's *age, work experience, gender*, and *professional belonging*, along with the *size of the team*, may influence leadership styles [13]. For instance, age and work experience may act as substitutes for leadership, i.e. the older and more experienced the staff, the less need they have for being supported by a leader [11,13]. Although research on gender differences in leadership style is not clearly conclusive [14–17], there seems to be a tendency for males to be more occupied with leadership in general than females, and slightly more oriented towards task and change styles, while females are slightly more relational oriented [1]. Empirical findings hint that organizational members belonging to the same profession will be more attentive to and more willing to accept the lead role of colleagues [18]. This implies that staff physicians may be more attentive to leadership styles in general than, for instance, support staff, because the majority of leaders in general practice are physicians. Finally, research has revealed that the size of an organization or the group being led may influence leadership style: the larger the organization or group, the more dominant and task oriented is the leader [1].

## Material and methods

This is a cross-sectional study using self-administrated questionnaires distributed by mail to 101 primary medical care facilities in Northern Norway, a predominantly rural region [19,20]. A total of 22 leadership style items were constructed on the basis of former scales identifying task, relation, and change styles and adjusted to our PHC context [1,11,12]. These items were ranked on Likert scales from 1, strongly disagree or at a very low degree, to 5, strongly agree or at a very high degree.

Altogether, 245 staff physicians and 350 support staff were invited and 127 and 222 respectively responded, which means that the response rate is 59%. Lead physician data were not included in the study, implying that leadership styles are identified solely through perceptions and expectations among employees (staff physicians and support staff).

Data were processed through factor analysis, univariate analysis, t-tests, ANOVA, Pearson product-moment correlation, and multivariate regression analyses. The selection of three components/ factors was done by using Kaiser's criterion using factors with an eigenvalue of 1.0 or more.

Staff physicians were 42% female and support staff 99.5% female. Staff physicians’ mean age was 39.6 years, and was slightly younger than the support staff (43.4 years, p < 0.001). Overall age ranged from 20 to 68 years. Average working experience at the centre differed considerably at 6.3 years for staff physicians’ and 11.6 years for support staff (p < 0.000). The average number of physicians at the health centres was 4.6, while the average number of support staff was 3.9.

## Results

**Figure 1.**
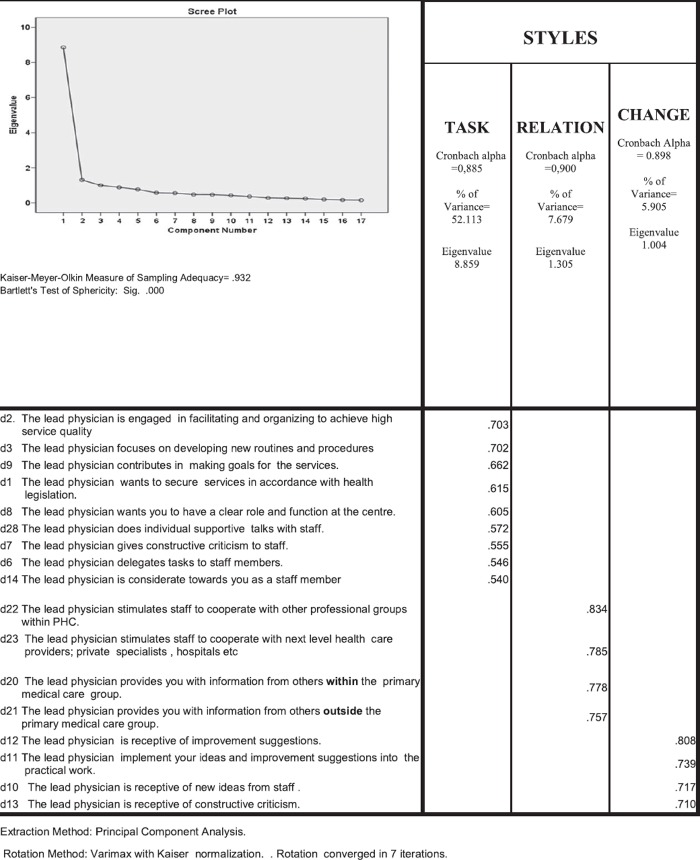
Result of factor analyses for initial 22 items. Notes: Screeplot included. Factor loadings, Cronbach's alpha and percentage of variance reported for each factor labelled style. Measures of sampling adequacy and test of sphericity are also indicated. Extraction method: principal component analysis. Rotation method: varimax with Kaiser normalization. Rotation converged in seven iterations.

Factor analyses were used to identify leadership styles. Principal component extraction rotated to a varimax criterion revealed three factors. Cronbach's alpha coefficient (α) was used as a reliability test. The data are presented in Figure 1.

The first factor, named here “task style”, loaded on items indicating that the leader is occupied with quality, routines, goals, and standards, and also showed interest in delegation. This factor also included dyadic relational behaviour such as emphasizing subordinate dialogue, giving subordinates constructive feedback, and being supportive and considerate towards staff. This style then incorporates items that in other studies are used to form a relational style.

The second factor, named here “relational style”, was formed by items indicating that the leader stimulates cooperation with local co-partners, cooperation with next-level institutions (hospital), and provides information to subordinates from within and from outside the health centre. Thus, this style is somewhat similar to what other studies have identified as a strategic leadership style [1].

The third factor, named “change style”, was constructed by items indicating that the lead physicians are focused on improvement suggestions and new ideas, implementation of new ideas, and receptiveness to constructive criticism. Thus, this factor is fairly similar to the former operationalization of this style [1].

### Descriptive

The mean style scores for all respondents, professions, and gender are reported in Table I.

**Table I.** Mean score distribution of styles within and between profession and gender groups.

Compare style means	Change style		Task style		Relation style	
	Mean score	Compared/p-values	Mean score	Compared/p-values	Mean score	Compared/p-values
1. All respondents (n = 329)	3.3558	Compared with task p < 0.000	3.1699	Compared with relation p < 0.000	2.8797	Compared with change p < 0.000
2. Staff physicians n = 119	3.5577	Compared with task p < 0.000	3.2147	Compared with relation n.s.	3.1928	Compared with change p < 0.000
3. Support staff n = 210	3.2484	Compared with task p < 0.01	3.1379	Compared with relation p < 0.000	2.6994	Compared with change p < 0.000
4. Staff physician, females n = 49	3.4583	Compared with task p < .000	2.9977	Compared with relation n.s.	3.0969	Compared with change p < .000
5. Professions Staff physicians (n = 119)	3.5360	Between professions p < 0.01	3.2113	Between professions n.s.	3.1928	Between professions p < 0.01
Support staff (n = 210)	3.2520		3.1386		2.6994	
6. Staff physicians by gender Female (n = 53)	3.4583	Between genders n.s.	3.0191	Between genders p < 0.05	3.0969	Between gender n.s.
Male (n = 73)	3.5870		3.3496		3.2500	

Note: n.s. = not significant.

The table presents mean style scores within profession and gender groups (rows 1–4) and scores between different profession and gender groups within each style (rows 5–6).

Analysis among all respondents revealed that change style scored highest (3.36) and relation style lowest (2.88). All three styles scored significantly differently (p < 0.000).

Among staff physicians the same distribution of styles was found, but scores were significantly different only between change and task style (p < 0.000). This is in contrast to the support staff group, which scored significantly differently for all styles (p < 0.01) though the style score ranking was the same.

### Correlation and regression analysis

In order to reveal what may cause the various leadership styles, we carried out bivariate and multivariate analyses. Bivariate analyses were carried out between key background and contextual variables and the three leadership styles for the whole sample. The results are displayed in Table II.

**Table II.** Means (M), standard deviations (SD), and Pearson correlations (n = 340).

	M	SD	1	2	3	4	5	6	7
1. Staff profession (Support staff = 0, Staff physicians = 1)	–	–	–						
2. Staff gender (female = 0 male = 1)	–	–	0.675**	–					
3. Staff age	42.03	10.09	–0.181**	–0.007	–				
4. Staff work experience	9.65	8.61	–0.294**	–0.137*	0.59**	–			
5. Team size	8.5	4.2	0.073	0.014	0.00	–0.06	–		
6. Relation style	2.88	1.04	0.229**	0.182**	–0.12*	–0.08	0.07	–	
7. Task style	3.17	0.86	0.041	0.113*	–0.06	–0.07	0.06	0.67**	–
8. Change style	3.36	0.97	0.141*	0.108*	–0.13*	0.11*	0.01	0.76**	0.63**

Notes: *Correlation is significant at 0.05 level (two-tailed). **Correlation is significant at 0.01 level (two-tailed). n is less than 349 because cases are excluded pair-wise in the analysis and some had not answered all questions.

The correlation analyses revealed that staff gender is significantly related to all the three leadership styles, which means that male staff are more associated with all leadership styles than females. Age is significantly negatively related to the relation style and the change style, which means that the older health care employees in all categories are, the less associated they are with these two styles. Work experience is significantly negatively related only to the change style.

A standard multiple regression analysis was performed for all staff and for staff physicians and support staff separately in order to identify factors that could predict the three leadership styles. Independent variables were age, work experience, team size, gender, and profession (see Table III).

**Table III.** Standard multiple regression for predicting leadership style.

	Change			Task			Relation		
	All (n = 330)	Physicians (n = 118)	Support (n = 210)	All (n = 335)	Physicians (n = 120)	Support (n = 211)	All (n = 329)	Physicians (n = 118)	Support (n = 209)
Age	–0.094	–0.281*	–0.048	–0.050	–0.098	–0.037	–0.122	–0.098	–0.143
Work experience	–0.025	0.132	–0.083	–0.044	–0.001	–0.073	0.051	0.072	0.032
Team size	0.004	0.078	–0.030	0.059	0.152	0.008	0.062	0.140	0.025
Gender (female = 0/male = 1)	0.047	0.131	–0.148*	0.176*	0.228*	0.009	0.076	0.103	–0.017
Profession (support staff = 0/staff physicians = 1)	0.084			–0.104			0.166*		
R^2^	0.032	0.049	0.033	0.025	0.063	0.010	0.067	0.030	0.018
F-value	2.017	1.354	1.690	1.605	1.840	0.499	4.407**	0.824	0.882

Notes: Standardized beta-coefficients are reported. *p < 0.05, **p < 0.01.

Age was a negative predictor of change style among physicians, which means that the older physicians are, the less need they have for leaders who are occupied with change and development tasks. Moreover, the results confirmed the importance of gender, as revealed through the correlation analyses. Among support staff, males seem to be less attentive to or in need of change style, although there are few males in this group. Males seem to be more attentive to or in need of task style than females in the sample as a whole and among physicians in particular. Also relation style seems to be predicted by gender, in the sense that males are significantly more attentive to or in need of this style. Neither work experience nor team size seems to have an influence on leadership styles in this sample.

## Discussion

This study asked what leadership styles could be found in rural general practice, what style dominates, and whether staff perceptions of styles may be due to their own professional belonging, gender, maturity in terms of age and experience, and team size.

For the whole study population, we identified three styles that were statistically highly different (see Figure 1). However, factor analyses revealed that the task style also included dimensions that traditionally have been subsumed under the relation style, for instance, emphasizing individual staff dialogue, giving constructive feedback, and being supportive and considerate towards staff. The explanation may be that the lead physician in rural general practice is also a clinician and integrated in the team. This facilitates and requires leadership where task- and problem-oriented styles are closely linked with individual relational behaviour.

In the sample as a whole, the change style was perceived the most. A reasonable explanation may be that general practice is challenged by demands from patients, the local community, and national health authorities for constantly better quality and efficiency, as well as demands from internal professional development for good practice. As medical care is undergoing reforms both locally and nationally, focus on change becomes a necessity [3–6]. Some interesting differences as to the presence of the styles between professions will be commented on (see Table I). When staff physicians seem to be more occupied with the change style than the support staff are, this could be explained by the fact that physicians, whether or not they occupy a leadership role, are heavily exposed to changes within their profession. This makes them more used to changes, and consequently more attentive to change processes in general. Also, the staff physician group has a superior position in the team and hence a specific responsibility for development processes. In addition, the work of the support staff is possibly characterized more as routine work, and could therefore be in less need of change and hence demands less change style from the leader.

Differences pertaining to the relation style between professions may be due to how this style is operationalized. In this study, the relation style focused on cooperation horizontally with other departments and institutions in the municipality as well as vertically with the next referral level. It also included information management along the same axes. This might be less relevant for the support staff. This professional difference is confirmed by the fact that there is no gender difference within the staff physician group on this style (Table I, row 6) as well as no physician gender difference between task style and relation style scores (Table I, row 4).

Both correlation and regression analyses show that there is a positive association between specifically male staff and all three styles. This could partly be explained by the general perceptions of leadership being associated with male values. The explanation for the strong link between male staff and perceived task style in particular could be found in the high congruence between this style's inclusion of managerial, directive, and problem-focused behaviour, and male values in general, such as emphasizing decisiveness and being results oriented [14,16,17]. This gender difference is specifically visible within the staff physician group (Table I, row 6).

Within the staff physician group, the strong link between male staff and task style was also demonstrated, though for change and relation styles, no gender difference was found (see Table I). The latter might indicate that when both gender groups belong to the same profession, and have the same role, responsibilities, and tasks to perform, it moderates differences in gender-based leadership style perceptions.

According to the results from correlation analyses, staff maturity in terms of age and work experience seems to co-vary negatively with change and relation styles (see Tables I and II). The explanation could be that age and work experience may be regarded as substitutes for these leadership styles [13].

Although research in general has shown that the larger the organization, the more task style is performed, our results displayed in Table II found no such associations. This could be due to the overall teams being fairly small [1].

Despite the low F-values in the regression analyses, which may reduce the generalizability of the results, our findings may still add to the understanding of leadership in primary health care organizations.

## Conclusion

The aim of this study was to identify leadership styles in primary medicine, how style perception is distributed between staff and gender, and, finally, how these styles are associated with and predicted by staff characteristics and team size.

In conclusion, our study confirmed that leadership in rural general practice can be identified in terms of change, task, and relation styles. Change style is perceived most by staff. However, due to the fact that leadership in general practice may differ from leadership in traditional organizations, we found that task style also included some relational aspects. This fact may explain why it received a significantly higher score than relational style.

Overall, males are clearly more attentive to leadership than females, specifically to task style. However, within the staff physician group, we see less difference between genders. Age and work experience (maturity) reduce the perception of and attention to leadership because these characteristics may reduce the need for leadership.

Since support staff scored lowest on all leadership style, but, at the same time, represent stability, and hence influence culture, it could possibly be a good idea to find out whether this is due to the leadership styles themselves, or the actual needs for leadership among these employees.

Taking into consideration the increasing focus on leadership in general practice due to demands for efficiency and change processes, an increasing number of young female physicians, and more diverse staff groups, these findings represent useful information for the understanding of leadership and a contribution to future training programmes.

## References

[1] Yukl G (2010). Leadership in organizations.

[2] National Directorate for Health and Social Affairs (2005). “… and it's going to get better!”– National Strategy for Quality Improvement in Health and Social Services (2005–2015).

[3] World Health Organization (2008). The World Health Report 2008: Primary health care now more than ever.

[4] Baker R (2000). Reforming primary care in England – again: Plans for improving the quality of care. Scand J Prim Health Care.

[5] Norwegian Medical Association http://www.legeforeningen.no/id/18.

[6] Hana J, Rudebeck CE (2011). Leadership in rural medicine: The organization on thin ice?. Scand J Prim Health Care.

[7] (2009). Report No. 47 (2008–2009) to the Storting. The coordination reform.

[8] Tharaldsen A (2011). Personal correspondence.

[9] Hemphill JK, Coons AE, Stogdill RM, Coons AE (1957). Development of the leader behavior description questionnaire. Leader behavior: Its description and measurement.

[10] Ekvall G, Arvonen J (1991). Change-centred leadership: An extension of the two-dimensional model. Scand J Management.

[11] Bass BM (1990). Handbook of leadership: A survey of theory and research.

[12] Hersey P, Blanchard KH (1977). The management of organizational behavior. 3rd ed.

[13] Kerr S, Jermier JM (1978). Substitutes for leadership: Their meaning and measurement. Organizational Behavior and Human Performance.

[14] Eagly AH, Johannesen-Schmidt MC, van Engen ML (2003). Transformational, transactional, and laissez-faire leadership styles: A meta-analysis comparing women and men. Psychol Bull.

[15] Anderson N, Lievens F, van Dam K, Born M (2006). A Construct-driven investigation of gender differences in a leadership- role assessment center. J Appl Psychol.

[16] Eagly AH, Carli LL (2003). The female leadership advantage: An evaluation of the evidence. Leadership Q.

[17] Eagly AH, Johnson BT (1990). Gender and leadership style: A meta-analysis. Psychol Bull.

[18] Conger JA, Kanungo RN (1987). Toward a behavioral theory of charismatic leadership in organizational settings. Academy of Management Rev.

[19] Lian OS, Merok E (2005). Mellom nostalgi og avant- garde. Distriktsmedisin i moderne tid [Between nostalgia and avant-garde. Rural medicine in modern times].

[20] Strasser R (2003). Rural health around the world: Challenges and solutions. Fam Pract.

